# Coupling Neurogenetics (GARS™) and a Nutrigenomic Based Dopaminergic Agonist to Treat Reward Deficiency Syndrome (RDS): Targeting Polymorphic Reward Genes for Carbohydrate Addiction Algorithms

**DOI:** 10.17756/jrds.2015-012

**Published:** 2015-06-24

**Authors:** Kenneth Blum, Thomas Simpatico, Rajendra D. Badgaiyan, Zsolt Demetrovics, James Fratantonio, Gozde Agan, Marcelo Febo, Mark S. Gold

**Affiliations:** 1 Department of Psychiatry & McKnight Brain Institute, University of Florida College of Medicine, Gainesville, FL, USA; 2 Department of Nutrigenomics, RD Solutions Inc., Salt Lake City, UT, USA; 3 Department of Addiction Research & Therapy, Malibu Beach Recovery Center, Malibu Beach, CA, USA; 4 Department of Psychiatry, Human Integrated Services Unit, University of Vermont Center for Clinical & Translational Science, University of Vermont College of Medicine, Burlington, VT, USA; 5 Department of Personalized Addiction Medicine IGENE, LLC, Austin, TX, USA; 6 Division of Applied Research & Education and Addition Services, Dominion Diagnostics, LLC., North Kingstown RI, USA; 7 Department of Nutrigenetic & Nutrigenomic Research, Victory Nutrition International, Austin, TX, USA; 8 Department of Personalized Medicine, Path Foundation, NY, USA; 9 Department of Psychiatry, University of Minnesota, College of Medicine, MN, USA; 10 Eotvos Lorand University, Institute of Psychology, Department of Clinical Psychology and Addiction, Izabella utca, Budapest, Hungary; 11 Departments of Psychiatry & Behavioral Sciences, Keck School of Medicine of USC, Los Angeles, CA, USA; 12 Department of Psychiatry, Washington University School of Medicine. St. Louis, MO, USA

**Keywords:** Nutrigenetics, Genetic Addiction Risk Score, Dopamine Agnostic therapy, Nutrigenomics, Personalized Addiction Medicine, Customized DNA based therapy

## Abstract

Earlier work from our laboratory, showing anti-addiction activity of a nutraceutical consisting of amino-acid precursors and enkephalinase inhibition properties and our discovery of the first polymorphic gene (Dopamine D2 Receptor Gene [DRD2]) to associate with severe alcoholism serves as a blue-print for the development of “Personalized Medicine” in addiction. Prior to the later genetic finding, we developed the concept of Brain Reward Cascade, which continues to act as an important component for stratification of addiction risk through neurogenetics. In 1996 our laboratory also coined the term “Reward Deficiency Syndrome (RDS)” to define a common genetic rubric for both substance and non-substance related addictive behaviors. Following many reiterations we utilized polymorphic targets of a number of reward genes (serotonergic, Opioidergic, GABAergic and Dopaminergic) to customize KB220 [Neuroadaptogen- amino-acid therapy (NAAT)] by specific algorithms. Identifying 1,000 obese subjects in the Netherlands a subsequent small subset was administered various KB220Z formulae customized according to respective DNA polymorphisms individualized that translated to significant decreases in both Body Mass Index (BMI) and weight in pounds. Following these experiments, we have been successfully developing a panel of genes known as “Genetic Addiction Risk Score” (GARSp_DX_)™. Selection of 10 genes with appropriate variants, a statistically significant association between the ASI-Media Version-alcohol and drug severity scores and GARSp_Dx_ was found A variant of KB220Z in abstinent heroin addicts increased resting state functional connectivity in a putative network including: dorsal anterior cingulate, medial frontal gyrus, nucleus accumbens, posterior cingulate, occipital cortical areas, and cerebellum. In addition, we show that KB220Z significantly activates, above placebo, seed regions of interest including the left nucleus accumbens, cingulate gyrus, anterior thalamic nuclei, hippocampus, pre-limbic and infra-limbic loci. KB220Z demonstrates significant functional connectivity, increased brain volume recruitment and enhanced dopaminergic functionality across the brain reward circuitry. We propose a *Reward Deficiency System Solution* that promotes early identification and stratification of risk alleles by utilizing GARS_Dx_, allowing for customized nutrigenomic targeting of these risk alleles by altering KB220Z ingredients as an algorithmic function of carrying these polymorphic DNA–SNPS, potentially yielding the first ever nutrigenomic solution for addiction and pain.

## Background

We are entering the era of genomic medicine and neuroimaging as it relates to addiction, a subset of Reward Deficiency Syndrome (RDS) [1]. In 2005, our laboratory received the first USA patent on Nutrigenomics and RDS treatment. This was awarded on the basis of our earlier work showing anti-addiction activity of a nutraceutical consisting of amino-acid precursors and enkephalinase inhibition properties and our discovery of the first polymorphic gene (Dopamine D2 Receptor Gene [DRD2] to associate with severe alcoholism [2]. Since that time the role of DRD2 gene and many of its variations have been confirmed by both NIDA and NIAAA scientists as well other investigators worldwide [3–6].

Prior to the later genetic finding, we developed the concept of Brain Reward Cascade, which continues to act as blue-print for stratification of addiction risk through neurogenetics [7]. In 1996, our laboratory also coined the term “Reward Deficiency Syndrome (RDS)” to define a common genetic rubric for both substance and non-substance related addictive behaviors [8]. At that time, we suggested that the dopaminergic system, and in particular the dopamine D2 receptor, has been profoundly implicated in reward mechanisms in the meso-limbic circuitry of the brain. Moreover, dysfunction of the D2 dopamine receptors leads to aberrant substance (alcohol, drug, tobacco and food) and other addictive seeking behaviors. Based on the Bayes Theorem, we found that the predictive value, in terms of subsequent seeking behaviors in carriers born with the DRD2 A1 allele, was 74.4%. Thus, we proposed that variants of DRD2 are important common genetic determinants in predicting compulsive disease. Almost two decades later we now know that RDS is a polygenic disorder due to impairments in the brain reward circuitry, especially with disruption of resting state functional connectivity. One novel example has recently been discovered by Stein’s group at NIDA [9] showing that cocaine addiction is associated with disturbed rsFC in striatal-cortical circuits. Specifically, compulsive cocaine abuse was associated with a balance of increased striatal-anterior prefrontal/orbit frontal and decreased striatal-dorsal anterior cingulate connectivity and trait impulsivity. Moreover, cocaine compulsive use was associated with increased dorsal striatal-dorsal lateral prefrontal cortex connectivity. Understanding these basic tenants, we can develop appropriate genetic predisposition risk as well as personalized medicine for the potential treatment of all RDS behaviors as a shared commonality for mental illness.

## Seeking Solutions to RDS

In terms of finding important therapeutic targets to treat addictive behaviors or RDS, the field has been fraught with many failed attempts to find a real solution to this enormous health related societal problem.

Blum et al. [10] has argued that one reason for only moderate success concerning FDA approved “Medical Assisted Treatment (MAT)” [11] related to the basic understanding that for the most part these approved medications for alcohol, opiates and nicotine but not cocaine, cannabis, nor any known addictive behaviors (gambling, eating disorders, hypersexuality etc.) favors the blocking of dopamine (DA) function instead of appropriate activation and induction of “dopaminergic homeostasis”. By incorporating the later we should find a better solution for RDS and as such we the authors are proposing “dopamine agonistic therapy” instead of long-term “dopamine antagonistic therapy” [12].

## Is Personalized Medicine the Answer?

Scientists have been pondering how to treat unwanted addictive behaviors for at least 40 years. Many scientists exploring the meso-limbic system have provided deep insight into the addictive brain and the neurogenetic mechanisms involved in man’s quest for happiness [13]. In brief, the site of the brain where one experiences feelings of well- being is the meso-limbic system. But, this system is functionally connected to Pre-frontal cortex and other brain regions [14]. The striatum, consisting of the nucleus accumbens (NAc), is the part of the brain that has been termed the “reward center.” The chemical messages include serotonin, enkephalins, GABA and dopamine, glutamate, cannabinoid, and acetylcholine, all working in concert to provide a net release of DA at the NAc. It is well known that genes control the synthesis, vesicular storage, metabolism, receptor formation and neurotransmitter catabolism [15]. The polymorphic-versions of these genes have certain variations which could lead to an impairment of the neurochemical events involved in the neuronal release of DA. As mentioned earlier, the cascade of these neuronal events has been termed “Brain Reward Cascade” [7].

A breakdown of this cascade will ultimately lead to a dysregulation and dysfunction of DA. Since DA has been established as the “pleasure molecule” and the “anti-stress molecule,” any reduction in function could lead to reward deficiency and resultant aberrant substance seeking thoughts and behaviors and a lack of wellness. Whether one accepts either the surfeit or deficit theories or even both the role of dopamine in terms of “liking” or wanting” is very important in developing therapeutic strategies to prevent relapse during periods of recovery [16, 17].

*Homo sapiens* physiology is motivationally programmed to drink, eat, have sex and desire pleasurable experiences to better ensure survival. Impairment in the mechanisms involved in these natural processes lead to multiple impulsive, compulsive, and addictive behaviors governed by genetic polymorphic antecedents we now term RDS. While there are a plethora of genetic variations at the level of Pre-Frontal Cortex-mesolimbic activity, polymorphisms of the serotonergic-2A receptor (5-HTT2a); endorphinergic (PENK), Opiate receptor(s) (Mu, Delta, Kappa), Cannabinoids (CB1, FAAA), Glutaminergic (NMDA) dopamine D2 receptor (DRD2), Gabaergic (GABA Beta-subunit), Mono-Amine-Oxidase (MOA) A) and the Catechol-o-methyl-transferase (COMT) genes, predispose individuals to excessive cravings and resultant aberrant thoughts and behaviors. We know that this proposed panel is certainly incomplete and should include over 600 genes. However, it is our proposal, that when it is complete it will serve a ‘blue-print’ for the future development of personalized medicine in the treatment of many behavioral addictions including obesity. Currently, our recent data supports the view that the following pathways are involved in the metabolic syndrome expression in any individual: energy production and regulation; stress management; reward cravings of the brain, neuro-endocrine system and metabolism; and immune system (including inflammation regulation) [18].

Previously Blum’s group hypothesized that genotyping certain known candidate genes would provide DNA-individualized customized nutraceuticals that may have significant influence on body re-composition by countering various genetic traits [19].

Along these lines obesity and related symptoms significantly aggravates type 2 diabetes, and both obesity and diabetes are influenced by the interaction of genes and environmental factors (epigenetics). Exploration of the current literature has identified a number of candidate genes to be associated with both of these disorders and include amongst many others, DRD2, methylenetetrahydrofolate reductase (MTHFR), serotonin receptor (5-HT2a), Peroxisome Proliferator-Activated Receptor gamma (PPAR-γ), and Leptin (OB) genes [20].

In light of these early hypothesis-generating studies, and a paucity of research, we set out to design a study to evaluate the process of DNA-customization of a nutritional solution for both wellness and weight management, the first of its kind in the literature.

## KB220z and DNA for Obesity

We hereby review the results of a number studies [21–23] whereby Blum’s Laboratory genotyped 1,058 subjects, and these subjects were administered KB220z [formerly LG8839, Recomposize, Genotrim] (a complex Neuroadaptagen nutraceutical-dl-phenylalanine, chromium, l-tyrosine other select amino-acids and adaptogens) based on polymorphic outcomes. In a small subset, simple *t*-tests comparing a number of parameters before and 80 days on the nutraceutical were performed.

The significant clinical outcomes are as follows: *weight loss* (*p* < 0.008); *sugar craving reduction* (*p* < 0.008); *appetite suppression* (*p* < 0.004); *snack reduction* (*p* <0.005); *reduction of late night binging* (*p* < 0.007); *increased perception of over-eating* (*p* < 0.02)]; increased energy (*p* < 0.004); enhanced *quality of sleep* (*p* < 0.02) and *increased happiness* (*p* < 0.02). Polymorphic correlates were obtained for a number of genes (PPAR gamma 2, MTHFR, 5-HT2a, and DRD2 genes) with positive clinical parameters tested in this study. Importantly, of all the outcomes and gene polymorphisms, only the DRD2 gene polymorphism (Al allele) had a significant Pearson correlation with days on treatment (*r*= 0.42, *p*= 0.045). This 2 fold increase is a very important genotype for compliance in treatment [21, 23, 24].

In addition, Blum’s group systematically evaluated the impact of polymorphisms of these five candidate genes as important targets for the development of a DNA-customized nutraceutical KB220z to combat obesity with special emphasis on body re-composition as measured by BMI [22]. A total of 21 individuals were evaluated in a preliminary investigational study of KB220z.

The experiment was based on the results of buccal swab genotyping of each subject, an individualized customized nutraceutical formula was provided as a function of measured gene polymorphisms of the five gene candidates assessed. At the initiation of the experiment and every two weeks subsequently, each subject completed a modified Blum-Downs OPAQuE Scale™ [Overweight Patient Assessment Questionnaire]. The alleles included the DRD2 Al; MTHFR C 677T; 5HT2a 1438G/A; PPAR-γProl2Ala and Leptin Ob1875 < 208bp. Pre- and post hoc analysis revealed a significant difference between the starting BMI and the BMI following an average of 41 days (28–70 d) of KB220z intake in the 21 individuals. The pre-BMI was 31.2 (weight/Ht^2^) compared to the post BMI of 30.4 (weight/Ht^2^) with a significance value of *P* < 0.034 (one tailed). Similarly the pre-weight in pounds (lb) was 183.52 compared to the post weight of 179 lb with a significance value of *P* < (0.047). They also found trends for reduction of late night snacking, carbohydrate craving reduction, reduction of stress, reduction of waist circumference. Moreover, in the 41 day period they found a trend in weight loss whereby 71.4% of subjects lost weight. Thus 15 out of 21 subjects lost weight with a *z*score of 2.4 and significance value of *P* < (0.02). In this group 53% lost on average over 2.5% of their starting weight. It is important to note that weight lost in this group was not the result of any of survival-defiant deprivation, stimulation, elimination or excessive exercise tactics, which are the most common tactics used by the vast majority of weight loss programs. Such tactics cause the most significant rebound weight loss-gain yo-yo trends observed in most weight loss programs over time; and that contribute to the obesity pandemic. Results in this study demonstrate to the contrary that weight lost in this group was due to systemic homeostatic corrections mediated by rebalancing the Brain Reward Cascade with the KB220Z nutrigenomic technology.

Other preliminary findings requiring extensive further investigation, using a Path Analysis [non-customized KB220z], also found important associations regarding anti-obesity related behaviors. In a one year cross sectional open trial study of 24 unscreened individuals utilization of oral KB220z variant resulted in the following benefits: stress reduction; sleep enhancement; increase in energy level; generalized wellbeing; reduction in cravings (sweets/carbs); improvement in mental focus/memory; improvement in blood sugar levels; reduction in food consumption; loss of inches around waist; loss of weight; reduction in blood pressure; improvement in workout performance; reduction in drug seeking behavior; reduction in hyperactivity; reduction in cholesterol levels [25].

## Future Perspectives

We hereby propose that the result of utilizing this natural dopaminergic activating [potentially DNA customized] approach over time should lead to neuronal DA release at the NAc, potentiating a proliferation of D2 receptors. We are encouraged by previous results utilizing KB220Z in terms of neuroimaging and qEEG studies [26–28] showing both enhanced resting state functional connectivity in abstinent heroin addicts [26] and regulation of widespread theta activity in the cingulate gyrus of abstinent psychostimulant abusers [27] as similar effects observed in alcoholics [28].

Other support in humans is derived from anti-craving effects observed in numerous peer reviewed published clinical trials including randomized double-blind placebo controlled studies [1]. In fact, animal gene therapy utilizing cDNA vectors of the DRD2 gene implanted into the NAc results in decreased alcohol and cocaine craving behavior [29–33].

We, the authors, are cognizant that dopaminergic activation in the long–term dopamine agonist therapy, instead of blocking dopamine [34], should be utilized to treat not only alcohol, cocaine, and nicotine cravings, but glucose craving and other known behavioral addictions (*e.g.* gambling [35], hypersexuality [36], etc.). Thus the coupling of genetic antecedents such as the *“Genetic Addiction Risk Score”* [37] and nutrition may be a very viable alternative approach for the treatment of obesity.

Other work from Blum’s laboratory developed a theoretical modeling study, in which they sought to evaluate health and economic implications of a nutrigenomic product for weight loss. Meshkin and Blum [38] constructed a nutrigenomic economic model by linking (1) published study data related to the efficacy of a product and/or ingredients, (2) validated clinical assessments that have already been tied to health economics data, and (3) data involving condition prevalence and overall cost of illness. In this theoretical model, we demonstrate that a DNA-customized nutraceutical positively reduces the cost of illness at the macroeconomic and microeconomic level based upon a cost-effectiveness and cost-benefit analysis. From this proposed model, they have forecasted the important prognostic health economic implications of a nutrigenomic intervention to demonstrate a theoretical model of nutrigenomic economics as it relates to obesity.

Many reiterations amounting to fifteen variant formulae, allowed Blum’s group [21–23] to utilize polymorphic targets of a number of reward genes (serotonergic, Opioidergic, GABAergic, and Dopaminergic) to customize KB220/KB220Z [Neuroadaptogen-amino-acid therapy (NAAT)] by specific algorithms. To reiterate, identifying over 1,000 obese subjects in the Netherlands, a subsequent small subset was administered various KB220Z formulae customized according to respective DNA polymorphisms individualized that translated to significant decreases in both BMI and weight in pounds [21–23].

Most recently, Blum’s laboratory along with Brett Haberstick, Andrew Smolen and others have successfully developed an unpublished panel of genes known as “Genetic Addiction Risk Score (GARSp_DX_)™. When they selected 10 genes with appropriate variants, a statistically significant association between the ASI-Media Version -alcohol and drug severity scores and GARSp_Dx_™ was found. This observation was found in 273 patients attending seven diverse treatment centers. This important association could potentially set the stage for early clinical identification of a predisposition and linked personalized medicine as a nutrigenomic solution for all RDS behaviors [38].

Most importantly, as pointed out earlier, Blum et al. [26] reported that well researched variant of NAAT-KB220Z [39], in abstinent heroin addicts, remarkably increased resting state functional connectivity. It was observed that this enhanced activation of resting state functional connectivity was observed in a putative network that included the dorsal anterior cingulate, medial frontal gyrus, nucleus accumbens, posterior cingulate, occipital cortical areas, and cerebellum.

In other unpublished rat work at the University of Florida, Febo, Blum and others, found that KB220Z significantly activates, above placebo, seed regions of interest including the left nucleus accumbens, cingulate gyrus, anterior thalamic nuclei, hippocampus, pre-limbic and infra-limbic loci. This response induced by KB220Z demonstrates significant functional connectivity, increased brain volume recruitment and enhanced dopaminergic functionality across the brain reward circuitry. This robust yet selective response implies clinical relevance.

In essence neuronutrient amino acid-based compositions of the KB220Z type will cause the synthesis of the brain reward neurotransmitters like serotonin and catecholamines, and through its effect on the enkephalins will by virtue of inhibiting GABA cause a significant release of dopamine in the nucleus accumbens. This constant release of therapeutic dopamine (anti-stress substance) occupies dopamine D2 receptors, especially in carriers of the A1 allele (low D2 receptors and high glucose craving), and overtime (possibly 6–8 weeks) effects mRNA transcription leading to potential proliferation of D2 and other dopamine type of receptors (balancing with D1 dopamine receptor type as well), thereby, reducing craving for carbohydrates. Among many genes, the dopamine D2 receptor gene is part of the human Obesity gene map [40].

Finally, understanding the evolutionary aspects of eating behavior and genetic antecedents such as the survival-based ‘thrifty’ gene [41] (inter-relationships of fasting/starvation and fat production), allows for a better ‘mouse trap’ that could impact obesity and eating disorders. Testing of these genomic principles by utilizing neuroimaging techniques may help lower the current obesity epidemic (Neurobesigenics) and redeem joy in victims of RDS.

## Conclusion

**Figure 1:**
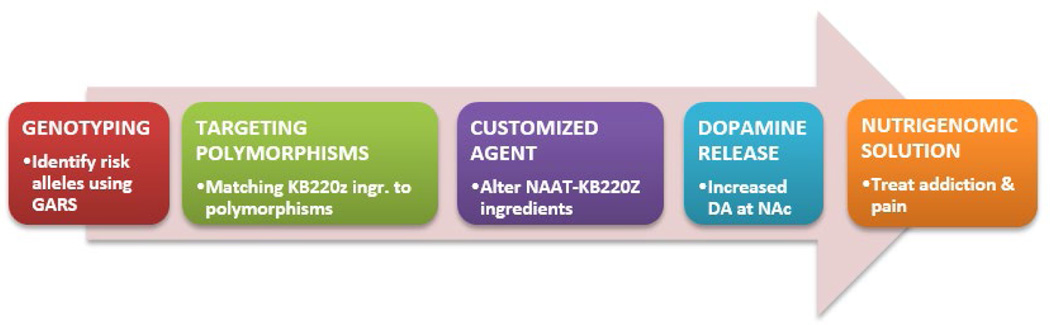
Nutrigenomic solution to RDS.

We the authors are now paused to propose a *Reward Deficiency System Solution™* that promotes early identification and stratification of risk alleles by utilizing GARSp_Dx_™, allowing for customized nutrigenomic targeting of these risk alleles by altering NAAT ingredients as an algorithmic function of carrying these polymorphic DNA–SNPS. By doing so, following required research, this novel approach could potentially yield the first ever nutrigenomic solution for addiction and pain (see Figure 1). Welcome to the new era of genomic addiction medicine [42].
